# Low CRB-65 Scores Effectively Rule out Adverse Clinical Outcomes in COVID-19 Irrespective of Chest Radiographic Abnormalities

**DOI:** 10.3390/biomedicines11092423

**Published:** 2023-08-30

**Authors:** Alexander Liu, Robert Hammond, Kenneth Chan, Chukwugozie Chukwuenweniwe, Rebecca Johnson, Duaa Khair, Eleanor Duck, Oluwaseun Olubodun, Kristian Barwick, Winston Banya, James Stirrup, Peter D. Donnelly, Juan Carlos Kaski, Anthony R. M. Coates

**Affiliations:** 1 School of Medicine, University of St Andrews, St Andrews KY16 9TF, UK; aql1@st-andrews.ac.uk (A.L.); rjhh@st-andrews.ac.uk (R.H.); pdd21@st-andrews.ac.uk (P.D.D.); 2 Royal Berkshire NHS Foundation Trust, Reading RG1 5AN, UK; kennethchan1@nhs.net (K.C.); dr.chuk@doctors.org.uk (C.C.); beckyljohnson@live.co.uk (R.J.); duaa_khair@hotmail.com (D.K.); eleanor.duck@gmail.com (E.D.); seun.r.olubodun@gmail.com (O.O.); kristianbarwick@outlook.com (K.B.); jimstirrup@hotmail.com (J.S.); 3 Royal Brompton Hospital, London SW3 6NP, UK; w.banya@rbht.nhs.uk; 4 Molecular and Clinical Sciences Research Institute, St George’s University of London, London SW17 0QT, UK; jkaski@sgul.ac.uk; 5 Institute of Infection and Immunity, St George’s University of London, London SW17 0QT, UK

**Keywords:** coronavirus disease 2019, CRB-65, chest X-ray, diagnostic performance, inflammatory markers, prognosis, risk stratification

## Abstract

**Background:** CRB-65 (**C**onfusion; **R**espiratory rate ≥ 30/min; **B**lood pressure ≤ 90/60 mmHg; age ≥ **65** years) is a risk score for prognosticating patients with COVID-19 pneumonia. However, a significant proportion of COVID-19 patients have normal chest X-rays (CXRs). The influence of CXR abnormalities on the prognostic value of CRB-65 is unknown, limiting its wider applicability. **Methods:** We assessed the influence of CXR abnormalities on the prognostic value of CRB-65 in COVID-19. **Results:** In 589 study patients (71 years (IQR: 57–83); 57% males), 186 (32%) had normal CXRs. On ROC analysis, CRB-65 performed similarly in patients with normal vs. abnormal CXRs for predicting inpatient mortality (AUC 0.67 ± 0.05 vs. 0.69 ± 0.03). In patients with normal CXRs, a CRB-65 of 0 ruled out mortality, NIV requirement and critical illness (intubation and/or ICU admission) with negative predictive values (NPVs) of 94%, 98% and 99%, respectively. In patients with abnormal CXRs, a CRB-65 of 0 ruled out the same endpoints with NPVs of 91%, 83% and 86%, respectively. Patients with low CRB-65 scores had better inpatient survival than patients with high CRB-65 scores, irrespective of CXR abnormalities (all *p* < 0.05). **Conclusions:** CRB-65, CXR and CRP are independent predictors of mortality in COVID-19. Adding CXR findings (dichotomised to either normal or abnormal) to CRB-65 does not improve its prognostic accuracy. A low CRB-65 score of 0 may be a good rule-out test for adverse clinical outcomes in COVID-19 patients with normal or abnormal CXRs, which deserves prospective validation.

## 1. Introduction

In patients with coronavirus disease 2019 (COVID-19), the development of clinical severity scoring systems is important in facilitating effective risk stratification [1,2,3,4]. CRB-65 is a risk score used to predict mortality in patients with community-acquired pneumonia and for deciding whether a patient requires treatment in a hospital setting or at home [5]. The CRB-65 score is easy to estimate, requires only simple observational parameters (confusion, respiratory rate, blood pressure, age of 65 years or older) and has been validated for use in both inpatient and outpatient settings [6]. Patients with community-acquired pneumonia and a CRB-65 score of 0 will likely be suitable for home treatment; those with a score of 1 or 2 should be considered for hospital referral; and patients with CRB-65 scores of 3 or 4 warrant urgent hospital admission [7]. CRB-65 can be used to assess patients at an early stage of presentation (prior to admission or within the initial emergency department triage setting), even before any blood tests or radiological investigations have been performed, to determine the clinical course of a patient with community-acquired pneumonia [5,6]. Recent reports also suggested an extrapolatory role for CRB-65 in the early risk stratification of patients presenting with acute COVID-19 pneumonia by highlighting the ability of this simple score to predict adverse clinical outcomes in this patient group [1,2,3,4].

In established clinical practice, the presence of chest X-ray (CXR) abnormalities provides radiological confirmation of pneumonia [8], which is also an independent risk factor for adverse clinical outcomes in COVID-19 patients [9,10,11,12]. Whilst CXR findings such as ground glass changes and consolidation are common in acute COVID-19 patients, a significant proportion of patients can also present with normal CXRs [10,13,14]. Therefore, for CRB-65 to be widely applicable in COVID-19, it needs to be functional both in patients with radiological evidence of pneumonia (i.e., abnormal CXRs) and in patients with normal CXRs. However, the influence of CXR abnormalities on the prognostic value of CRB-65 is currently unknown, which hinders its potential clinical translation.

In this study, we sought to fill this knowledge gap by validating the prognostic value of CRB-65 in COVID-19 patients with normal and abnormal CXR findings. As a secondary study objective, we also compared the performance of CRB-65 (a non-serum-based biomarker) against established serum inflammatory biomarkers of infection (C-reactive protein (CRP) and white cell count (WCC)) for prognosticating COVID-19 patients. We hypothesised that the presence of CXR abnormalities significantly affects the ability of CRB-65 to predict prognosis in COVID-19 patients.

## 2. Materials and Methods

### 2.1. Study Subjects

This study included consecutive hospitalised patients with acute COVID-19 at the Royal Berkshire NHS Foundation Trust (a UK general hospital) between 5 March 2020 and 9 May 2020. Patients were included if they were aged 18 years or older, had COVID-19 confirmed by laboratory reverse transcription polymerase chain reaction (rt-PCR) testing of nasopharyngeal swab samples and underwent both CXR and serum inflammatory marker assessment upon presentation to hospital. Patients were excluded according to the following criteria: missing, incomplete or unclear follow-up data (*n* = 8); incomplete CRP or WCC data for comparison (*n* = 9); or treatment at another hospital prior to admission (*n* = 2). The final study population consisted of 589 patients.

### 2.2. Ethical Approval

This study received COVID-19 Fast-Track Approval from the Health Research Authority (HRA) and Health and Care Research Wales (HCRW), Cardiff, UK.

### 2.3. Data Collection

Patient demographic and clinical information, laboratory blood test results and CXR findings were collected by a group of study investigators from the electronic patient records. The data collection was performed in accordance with a pre-defined and standardised protocol. Each investigator first collected a training dataset of ten cases which were validated independently for accuracy against the source electronic patient records by another observer. After completing the data training process successfully, the investigators then collected the remaining data. To ensure data accuracy, after all data were collected, selections of data were independently checked by two observers as referenced to the source electronic patient records. Full anonymisation of the dataset then took place for statistical analysis.

### 2.4. Study Endpoints

The primary endpoint in the study was inpatient mortality attributed to acute COVID-19. For secondary endpoints, two were defined: (i) the need for non-invasive ventilation (NIV) due to acute COVID-19; and (ii) critical illness related to acute COVID-19, defined as a composite of a need for airway intubation and/or mechanical ventilation and/or admission to an intensive care unit (ICU).

CRB-65 scores (range: 0 to 4) were derived, as previously described [3,6,15], using the following parameters: confusion scores 1 point, finding of a respiratory rate of ≥30/min scores 1 point, having a systolic blood pressure ≤ 90 mmHg and/or a diastolic blood pressure ≤ 60 mmHg scores 1 point and age ≥ 65 years scores 1 point. CXR findings were reported by a radiologist as part of routine clinical care and thus blinded to this study.

### 2.5. Statistical Analysis

Continuous variables were checked for normality using the Kolmogorov–Smirnov test [16]; all continuous variables were shown to reject normality (all *p* < 0.05). Therefore, all continuous variables were expressed as median (inter-quartile range (IQR)). Continuous variables were compared using the Mann–Whitney test [17]. Categorical variables were compared using the chi-squared test [18]. The diagnostic performance of CRB-65 and other variables for predicting study endpoints was assessed using receiver operator characteristics (ROC) analysis [19]. Where appropriate, the areas under the ROC curves were displayed with standard error of the mean (SEM). Inpatient survival was assessed using Kaplan–Meier curves and compared using the logrank test [20], with events censored to 60 days to assess for 60-day inpatient survival. A Cox proportional hazard regression multivariate model with stepwise variable selection was utilised to assess for independent predictors of inpatient mortality [21]. Variables included CRB-65, CXR, CRP and WCC. The hazard ratios for the predictors of mortality were displayed with 95% confidence intervals (CI); *p*-values < 0.05 were considered statistically significant. The statistical analysis is this study was performed by the first author, A.L. (MedCalc, Version 20.104, Ostend, Belgium), and independently validated by a medical statistician, W.B. (Stata; Basic Edition version 17.0, Statacorp LLC, College Station, TX, USA).

## 3. Results

### 3.1. Baseline Characteristics

In the 589 COVID-19 patients in the study (median age of 71 (57–83); 57% males), 186 patients (32%) had normal CXRs and 403 patients (68%) had abnormal CXRs (Table 1). Patients with abnormal CXRs had a higher prevalence of dyspnoea and cough compared to patients with normal CXRs. Both patient groups had a similar burden of co-morbidities and medication history (Table 1).

### 3.2. Clinical Observations, Investigations and Clinical Outcomes

Patients with abnormal CXRs had slightly higher temperature and respiratory rates on admission compared to patients with normal CXRs (Table 2). The frequency of patients who were confused on presentation was similar in those with abnormal and normal CXRs (Table 2). The two patient groups also have similar levels of systolic and diastolic blood pressure (Table 2).

In patients with abnormal CXRs, ground glass and interstitial opacification were the commonest abnormalities (49%), followed by consolidation (29%), atelectasis (13%) and pleural effusions (11%; Table 2). Patients with abnormal CXRs had higher CRP levels (116 mg/L (52–224) vs. 64 mg/L (23–143); *p* < 0.001) and lower lymphocyte counts (0.89 × 10^9^/L (0.58 × 10^9^–1.30 × 10^9^) vs. 0.98 × 10^9^/L (0.69 × 10^9^–1.39 × 10^9^); *p* = 0.028, Table 2) compared to patients with normal CXRs. Inpatient mortality, NIV requirement and critical illness were more common in patients with abnormal CXRs than patients with normal CXRs (all *p* < 0.01, Table 2).

### 3.3. Distribution of CRB-65 Scores

**Figure 1:**
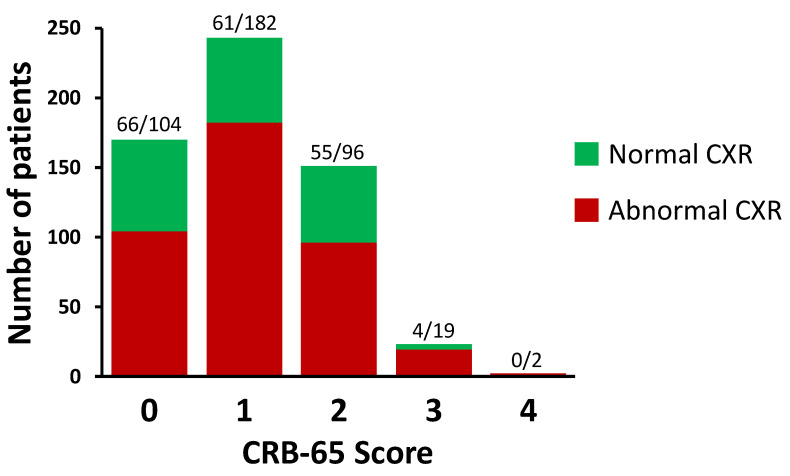
Distribution of CRB-65 scores in the study. The numbers of patients are shown above each bar as stratified by normal and abnormal chest X-rays (CXRs), respectively. A total of 589 patients were included in this study.

The distribution of CRB-65 scores in the study is shown in Figure 1. Most patients (96%) had CRB-65 scores between 0 and 2. Normal CXRs were found in 39% of patients with a CRB-65 score of 0; in 25% of patients with a CRB-65 score of 1; in 36% of patients with a CRB score of 2; and in 17% of patients with a CRB-65 score of 3. No patients with a CRB-65 score of 4 had normal CXRs.

### 3.4. Prognostic Value of CRB-65

**Figure 2:**
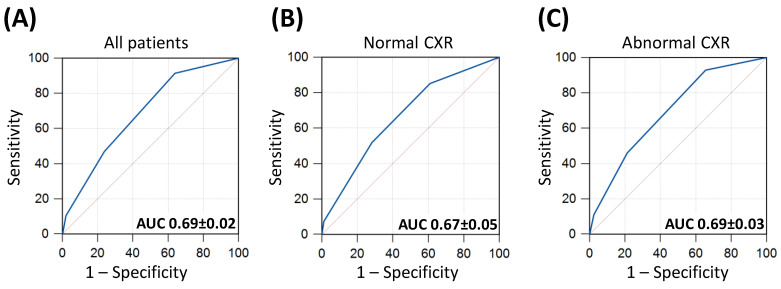
Receiver operator characteristics (ROC) curves of CRB-65 for predicting inpatient mortality. Panel (**A**) included all patients in the study; panel (**B**) included only patients with normal chest X-rays (CXRs) and panel (**C**) included only patients with abnormal CXRs. Areas under the ROC curves (AUC) are illustrated ± standard errors of the mean (SEM).

On ROC analysis, an optimal (Youden point) cut-off CRB-65 score of 0 achieved a sensitivity of 92% (95% CI: 86–95) and a specificity of 36% (95% CI: 32–41) for predicting inpatient mortality (AUC 0.69 ± 0.02, *p* < 0.001, Figure 2A). CRB-65 achieved similar diagnostic performance for predicting mortality in patients with normal CXRs and abnormal CXRs (AUC 0.67 ± 0.05 vs. 0.69 ± 0.03; *p* = 0.732, respectively; Figure 2).

The full range of diagnostic performance values of CRB-65 for predicting clinical outcomes is shown in Supplementary Tables S1 and S2.

In the whole study population, a low CRB-65 score of 0 achieved a negative predictive value (NPV) of 92% for ruling out inpatient mortality; an NPV of 87% for ruling out NIV requirement; and an NPV of 90% for ruling out critical illness. In patients with normal CXRs, a CRB-65 score of 0 achieved an NPV of 94% for ruling out mortality; an NPV of 98% for ruling out NIV requirement; and an NPV of 99% for ruling out critical illness. In patients with abnormal CXRs, a CRB-65 score of 0 achieved an NPV of 91% for ruling out mortality; an NPV of 83% for ruling out NIV requirement; and an NPV of 86% for ruling out critical illness.

In the whole study population, an intermediate CRB-65 score of 1 achieved an NPV of 80% for ruling out mortality; an NPV of 86% for ruling out NIV requirement; and an NPV of 92% for ruling out critical illness. A high CRB-65 score (2 or 3) had poor positive predictive values for predicting the occurrence of clinical outcome endpoints in the study.

### 3.5. Survival Curve Analysis

**Figure 3:**
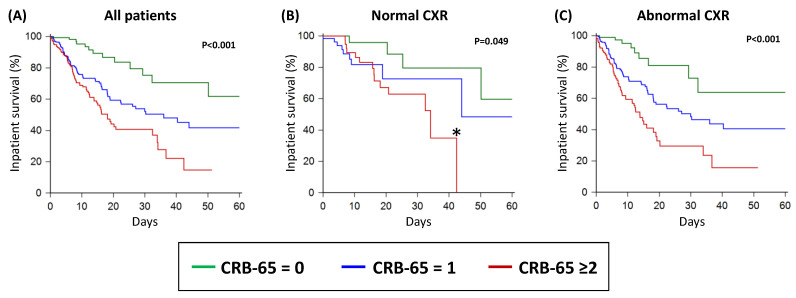
Inpatient survival of acute COVID-19 patients as stratified by the CRB-65 score in all patients (**A**); in patients with normal CXR (**B**); and in patients with abnormal CXR (**C**). CXR: chest X-ray. * There were no patients with normal CXRs and CRB-65 ≥ 2 after 42 days of follow up, which explains the apparent drop-off in the Kaplan–Meier curve; this apparent drop-off in the curve does not indicate that all patients died or were discharged alive.

By Kaplan–Meier analysis, as the CRB-65 score increased from 0 to 1 to ≥2, there were stepwise reductions in the inpatient survival within the whole study population (Figure 3A). A similar pattern was observed in patients with abnormal CXRs (Figure 3C). In patients with normal CXRs, those with CRB-65 scores of 0 and 1 had similar inpatient survival, which was significantly better than patients with CRB-65 scores ≥ 2 (Figure 3B).

### 3.6. Multivariate Analysis

Using a Cox proportional hazard regression analysis, only elevated CRB-65 ≥1 (hazard ratio of 3.75, 95% CI: 2.12–6.63; *p* < 0.001), abnormal CXR (hazard ratio of 1.86, 95% CI: 1.22–2.82; *p* = 0.004) and significantly elevated CRP > 120 mg/L (hazard ratio of 1.48, 95% CI: 1.06–2.07; *p* = 0.021) were predictors of inpatient mortality (Table 3).

### 3.7. Prognostic Value of CRB-65 Compared to Serum Inflammatory Markers

**Figure 4:**
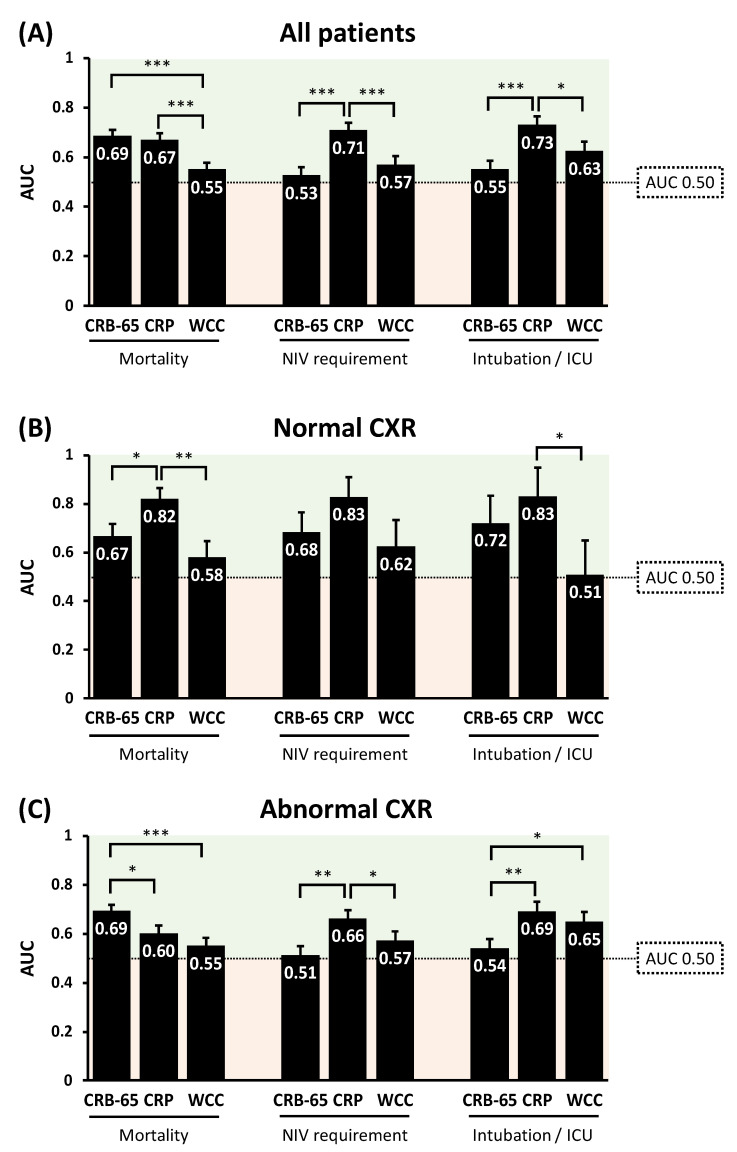
Comparisons of the diagnostic performance of CRB-65 and serum inflammatory biomarkers for predicting adverse clinical outcomes in COVID-19 patients, in all patients (**A**); in patients with normal CXR (**B**); and in patients with abnormal CXR (**C**). AUC: area under receiver operator characteristics curves; CRP: C-reactive protein; CXR: chest X-ray; ICU: intensive care unit; WCC: white cell count. * *p* < 0.05; ** *p* < 0.01; *** *p* < 0.001.

CRB-65 performed similarly to CRP for predicting inpatient mortality in the whole study population (Figure 4). Both CRB-65 and CRP outperformed WCC for predicting mortality (both *p* < 0.001; Figure 4). In patients with abnormal CXRs, CRB-65 outperformed CRP for predicting mortality (*p* < 0.05; Figure 4). However, in patients with normal CXRs, CRP outperformed CRB-65 for the same purpose (*p* < 0.05; Figure 4). For predicting NIV requirement and critical illness, CRP outperformed CRB-65 and WCC, irrespective of CXR findings (Figure 4).

## 4. Discussion

This study examined the influence of CXR abnormalities on the prognostic value of CRB-65 in COVID-19 patients, as compared to serum inflammatory markers. The main findings are: (i) adding CXR findings does not significantly improve the predictive power to the CRB-65 score; (ii) a low CRB-65 score of 0 effectively ruled out adverse clinical outcomes in COVID-19 patients with both normal and abnormal CXRs; (iii) a high CRB-65 score (2–3) did not accurately predict clinical outcomes in this study population—the course could still be benign, but may warrant greater clinical vigilance; (iv) CRB-65 outperformed established serum inflammatory markers (CRP and WCC) for predicting inpatient mortality in patients with abnormal CXRs; (v) CRB-65 was inferior to CRP for predicting NIV requirement and occurrence of critical illness; and (vi) COVID-19 sufferers with low CRB-65 scores have better inpatient survival than patients with high CRB-65 scores, without significant influence from the presence or absence of CXR abnormalities.

### 4.1. CRB-65 as a Rule-Out Test for Adverse Clinical Outcomes in COVID-19

The study findings suggest that the major utility of CRB-65 in the risk stratification of COVID-19 patients may be as a rule-out test for adverse clinical outcomes to identify a low-risk patient group, irrespective of major influences from the presence or absence of CXR abnormalities. In the absence of CXR abnormalities, a low CRB-65 score of 0 almost completely ruled out any adverse clinical outcomes based on this retrospectively studied population. CRB-65 appears to be a weak positive predictor of adverse clinical outcomes, and the data do not support the use of a high CRB-65 score in isolation for identifying high-risk COVID-19 patients.

The stage is now set to prospectively validate the rule-out utility of CRB-65 for facilitating hospital admission avoidance and possible early discharge of low-risk patients.

### 4.2. Logistical Advantages of CRB-65

A major advantage of CRB-65 lies in its simplicity, being derivable using routine parameters from clinical history and examination alone, which are familiar to most healthcare services worldwide. The fact that medical staff can work out whether a patient is confused, count the respiratory rate, take a blood pressure reading and find out the age of the patient within a few minutes of meeting a patient means that the CRB-65 score can provide a rapid (“on the spot”) assessment of clinical risk. Moreover, CRB-65 is arguably cheaper to perform since it does not require any blood tests nor imaging markers to be available.

### 4.3. Comparison of CRB-65 with Serum Inflammatory Markers

The sample size in this study is smaller than some of the other studies [22]; an important factor was that this sample size was adequate to test the hypothesis set out for the study; 589 patients were consecutive patients admitted to hospital within a consistent period during the pandemic. The mortality rate was 26% (153 out of 589 patients died), which offers an adequate level of primary endpoints for us to study the effect of the variables on mortality. The dataset for the 589 patients was also complete in terms of all patients having the investigative variables performed, including all components of CRB-65, CXR, CRP and WCC. To this end, the sample size was an adequate test bed for the study hypothesis.

The results of the multivariate analysis support the notion that CRB-65 is independent from CXR when it comes to predicting inpatient mortality. Both elevated CRB-65 scores and abnormal CXRs carried a higher risk of death in COVID-19 patients.

This study population also showed that in terms of diagnostic performance on ROC analysis, CRB-65 was, overall, comparable to CRP for predicting inpatient mortality in COVID-19 patients. However, unlike CRP, CRB-65 does not require any blood test to be performed. Furthermore, the finding that CRB-65 outperformed CRP for mortality prediction in patients with abnormal CXRs is interesting. It may be that CRB-65 assesses a range of clinical manifestations of the inflammatory response in patients with CXR changes, as compared to CRP, which is a relatively non-specific serum biomarker alone [23,24]. This possible mechanism deserves further investigation.

In patients with normal CXRs, CRP was superior to CRB-65 for predicting mortality. This finding is difficult to explain, though CRP may be able to detect underlying inflammatory response, which does not manifest in the abnormal clinical parameters that CRB-65 relies on. This possible concept also requires further characterisation.

The ability of a low CRB-65 score to rule out adverse complications in COVID-19 patients may lie in its potential to identify low-risk patients, which appears to be an inherent property of risk stratification biomarkers such as cardiac troponin [25] or D-dimer [26,27]. In the case of CRB-65, a low score potentially reflects a lack of systemic manifestation of clinical infection or a septic response to infection, which are indicators of prognosis [28]. Again, CRB-65 may be advantageous over serum biomarkers since CRB-65 does not require laboratory testing, thus avoiding potential delays and any added cost of blood sample testing [3,6].

CRB-65 performed poorly in relation to CRP for predicting NIV requirement, intubation and ICU admissions. This is a major disadvantage of CRB-65 and limits its use as a biomarker to anticipate the occurrence of non-fatal adverse complications in COVID-19. Although the underlying mechanism for this observation remains unclear, one explanation may be that the selection of patients for NIV, intubation and ICU admissions are multi-factorial and are not wholly based on changes in the clinical and observational parameters that CRB-65 relies on [27]. In fact, parameters such as increased respiratory rate and hypotension at presentation do not necessarily lead to further clinical deterioration [27]. Moreover, development of confusion is also multi-factorial rather than exclusively related to respiratory failure requiring NIV or intubation [29,30]. Although older age is linked to clinical deterioration in COVID-19 [27], it does not necessitate that all elderly patients would require NIV, intubation or ICU admission. Further work is required to elucidate the apparent disconnect between CRB-65 and non-fatal endpoints in COVID-19 patients. 

### 4.4. Limitations and Future Directions

This study has several limitations that could act as catalysts for future work. The retrospective and single-centred nature of the study means that the results may be prone to selection bias, which drives the need for a prospective and multi-centred study to further test the clinical value of CRB-65. The retrospective nature of the study also limited the completeness of certain datasets, and we were unable to include other clinical risk estimation systems, such as the 4C score [31], for comparison. This limitation also drives the need for a prospective validation study. This study focused on inpatient mortality of acute COVID-19 patients as the primary prognostic endpoint. Further studies that broaden the scope to include long-term morbidity, post-hospitalisation quality of life and persistent symptoms of long COVID-19 could more comprehensively address the natural history of the illness. Not all patients underwent chest computed tomography (CT) imaging, which may have provided more detailed assessment of radiological abnormalities associated with COVID-19. However, in clinical practice, only a minority of all patients presenting with COVID-19 would undergo chest CT scanning; therefore, the reliance on CXR for radiological diagnosis of pneumonia in this study is representative of standard clinical practice in the pandemic. The dynamic evolution of the individual components of CRB-65 such as respiratory rate and blood pressure or subsequent development of post-hospitalisation confusion could not be assessed, which may have important additional prognostic value over single-snapshot assessments on admission. The effect of secondary bacterial infections and therapies instigated for COVID-19 could not be assessed. In future research, integrating artificial intelligence methods such as deep convolutional neural networks (CNN) [32,33] with CRB-65 might offer value in prognosticating COVID-19 patients. Finally, this study was conducted at a time before routine vaccination and availability of many contemporary therapies for COVID-19. Nevertheless, the results of the study mean that CRB-65 could potentially be used as a new endpoint measure for future studies in a more contemporary patient population.

## 5. Conclusions

CRB-65, CXR and CRP are independent predictors of mortality in COVID-19. Adding CXR findings (dichotomised to either normal or abnormal) to CRB-65 does not improve its prognostic accuracy. A low CRB-65 score of 0 may be a good rule-out test for adverse clinical outcomes in COVID-19 patients with normal or abnormal CXRs, which deserves prospective validation.

## Figures and Tables

**Table 1:** Baseline characteristics.

	All Patients (*n* = 589)	Normal CXR (*n* = 186)	Abnormal CXR (*n* = 403)	*p*-Value
Demographics				
Age (years)	71 (57–83)	73 (56–85)	71 (58–82)	0.512
Male (%)	337 (57)	101 (54)	236 (59)	0.331
BMI (kg/m^2^)	26 (22–30)	25 (21–28)	27 (23–31)	<0.001
Symptoms				
Dyspnoea	328 (56)	80 (43)	248 (62)	<0.001
Cough	342/402 (58)	88/185 (48)	254 (63)	<0.001
Fever	289/402 (49)	84/185 (45)	205 (51)	0.219
Fatigue	143/402 (24)	39/185 (20)	104 (26)	0.215
Chest pain	67 (11)	23/185 (12)	44 (11)	0.592
Comorbidities				
Hypertension	272 (46)	79 (42)	193 (48)	0.220
Current/Ex-Smoker	174 (30)	51 (27)	123 (31)	0.443
Diabetes	168/584 (29)	49/183 (27)	119/401 (30)	0.492
CKD	142 (24)	41 (22)	101 (25)	0.473
Atrial fibrillation	98/585 (17)	26/183 (14)	72/402 (18)	0.266
IHD	88/587 (15)	27 (15)	61/401 (15)	0.820
Asthma	73/588 (12)	24 (13)	49/402 (12)	0.807
COPD	71/588 (12)	17 (9)	54/402 (13)	0.137
Dyslipidaemia	69/587 (12)	22 (12)	47/401 (12)	0.970
Heart failure	65/587 (11)	19 (10)	46/401 (11)	0.652
CVA/TIA	65 (11)	25 (13)	40 (10)	0.206
Medications				
ACEi/ARB	153/588 (26)	40 (22)	113/402 (28)	0.090
Statins	196/588 (33)	52 (28)	144/402 (36)	0.060
Beta Blockers	143/588 (24)	38 (20)	105/402 (26)	0.135
Aspirin	77/587 (13)	17/185 (9)	60/402 (15)	0.056

ACEi: angiotensin-converting enzyme inhibitor; ARB: angiotensin receptor blocker; BMI: body mass index; CKD: chronic kidney disease; COPD: chronic obstructive pulmonary disease; CVA: cerebrovascular accident; IHD: ischaemic heart disease; TIA: transient ischaemic attack.

**Table 2:** Clinical observations, investigations and clinical outcomes.

	**All Patients** **(*n* = 589)**	**Normal CXR** **(*n* = 186)**	**Abnormal CXR** **(*n* = 403)**	***p-*** **Value**
Age (years)	71 (57–83)	73 (56–85)	71 (58–82)	0.512
Observations				
Confused on presentation	80 (14)	31 (17)	49 (12)	0.138
Temperature (°C)	37.1 (36.6–37.9)	36.9 (36.6–37.6)	37.1 (36.6–37.9)	0.031
SBP (mmHg)	128 (114–145)	129 (114–146)	128 (113–144)	0.739
DBP (mmHg)	74 (66–82)	74 (67–82)	74 (65–83)	0.930
Respiratory Rate (/min)	21 (18–25)	20 (18–22)	22 (19–28)	<0.001
CXR abnormalities				
GGO	196/571 (34)	-	196/396 (49)	-
Consolidation	113/569 (20)	-	113/394 (29)	-
Atelectasis	52/571 (9)	-	52/396 (13)	-
Pleural effusions	43/570 (8)	-	43/395 (11)	-
Laboratory Results				
Haemoglobin (g/L)	125 (109–142)	127 (110–143)	125 (108–142)	0.354
WCC (10^9^/L)	7.6 (5.6–10.5)	7.5 (5.7–11.2)	7.6 (5.5–10.3)	0.442
Lymph. Count (×10^9^/L)	0.90 (0.60–1.31)	0.98 (0.69–1.39)	0.89 (0.58–1.30)	0.028
Platelet Count (10^9^/L)	224 (174–291)	221 (174–293)	226 (174–290)	0.742
CRP (mg/L)	101 (41–198)	64 (23–143)	116 (52–224)	<0.001
Sodium (mmol/L)	138 (134–140)	138 (135–140)	138 (134–140)	0.339
Potassium (mmol/L)	4.2 (3.8–4.6)	4.2 (3.8–4.5)	4.2 (3.9–4.6)	0.577
Creatinine (μmol/L)	88 (67–134)	86 (65–117)	89 (68–136)	0.206
Complications				
Inpatient mortality	153 (26)	27 (15)	126 (31)	<0.001
NIV requirement	83 (14)	9 (5)	74 (18)	<0.001
Intubation/ventilation	36 (6)	3 (2)	33 (8)	0.001
ICU admission	65 (11)	6 (3)	59 (15)	<0.001

CRP: C-reactive protein; CXR: chest X-ray; DBP: diastolic blood pressure; ICU: intensive care unit; GGO: ground glass opacification; NIV: non-invasive ventilation; SBP: systolic blood pressure; WCC: white blood cell count.

**Table 3:** Multivariate analysis of independent mortality predictors in COVID-19 patients.

	**Hazard Ratio (95% CI)**	***p*-Value**
CRB-65 ≥ 1	3.75 (2.12–6.63)	<0.001
Abnormal CXR	1.86 (1.22–2.82)	0.004
CRP	1.48 (1.06–2.07)	0.021

CI: confidence interval; CRP: C-reactive protein; CXR: chest X-ray.

## Data Availability

The terms of the ethical approval do not allow the data to be put in a public domain, nor for the open sharing of patient specific clinical data even when anonymized. Data access requests may be sent to the Health Research Authority (HRA) and Health and Care Research Wales (HCRW) at approvals@hra.nhs.uk.

## Supplementary Materials

The following supporting information can be downloaded at: https://www.mdpi.com/article/10.3390/biomedicines11092423/s1, Table S1: Diagnostic performance of low CRB-65 score for predicting adverse clinical outcomes; Table S2: Diagnostic performance of intermediate to high CRB-65 scores for predicting adverse clinical outcomes.
