# Supplementary material for: Low CRB-65 Scores Effectively Rule out Adverse Clinical Outcomes in COVID-19 Irrespective of Chest Radiographic Abnormalities
Source: Biomedicines. 2023 Aug 30;11(9):2423. doi: 10.3390/biomedicines11092423 (PMC10525183; doi:10.3390/biomedicines11092423)
Supplement: Supplementary file 1 [file biomedicines-11-02423-s001.zip › biomedicines-2561429-supplementary.pdf]

Supplementary materials

Supplementary Table S1. Diagnostic performance of low CRB-65 score for predicting adverse clinical outcomes.

	N	AUC ±SEM	*p* Value	CRB-65 Cut-off	Sensitivity (95% CI)	Specificity (95% CI)	Positive LR (95% CI)	Negative LR (95% CI)	PPV (95% CI)	NPV (95% CI)
**Mortality**										
All patients	589	0.69±0.02	<0.001	0	92% (86-95)	36% (32-41)	1.4 (1.3-1.6)	0.2 (0.1-0.4)	33% (32-35)	92% (88-95)
Normal CXR	186	0.67±0.05	0.002	0	85% (66-96)	39% (31-47)	1.4 (1.1-1.7)	0.4 (0.2-1.0)	19% (16-23)	94% (86-98)
Abnormal CXR	403	0.69±0.03	<0.001	0	93% (87-97)	34% (29-40)	1.4 (1.3-1.6)	0.2 (0.1-0.4)	39% (37-42)	91% (85-95)
**NIV requirement**										
All patients	589	0.53±0.03	0.416	0	35% (25-46)	72% (68-76)	1.3 (0.9-1.7)	0.9 (0.8-1.1)	17% (13-22)	87% (85-89)
Normal CXR	186	0.68±0.08	0.026	0	67% (30-93)	66% (59-73)	2.0 (1.2-3.3)	0.5 (0.2-1.3)	9% (6-14)	98% (94-99)
Abnormal CXR	403	0.51±0.04	0.711	0	31% (21-43)	75% (70-80)	1.3 (0.9-1.9)	0.9 (0.8-1.1)	22% (16-30)	83% (81-85)
**Intubation / ICU**										
All patients	589	0.55±0.03	0.125	0	34% (23-47)	72% (68-76)	1.2 (0.8-1.7)	0.9 (0.8-1.1)	13% (9-18)	90% (88-91)
Normal CXR	186	0.72±0.11	0.047	0	83% (36-100)	66% (59-73)	2.5 (1.6-3.7)	0.3 (0.0-1.5)	8% (5-11)	99% (95-100)
Abnormal CXR	403	0.54±0.04	0.239	0	29% (18-42)	75% (70-79)	1.1 (0.7-1.8)	1.0 (0.8-1.1)	16% (11-23)	86% (84-88)

AUC: area under the receiver operator characteristics curve; CI: confidence interval; CXR: chest X-ray; ICU: intensive care unit admission; LR: likelihood ratio; N: number of acute COVID-19 patients; NIV: non-invasive ventilation; NPV: negative predictive value; PPV: positive predictive value; SEM: standard error of the mean.

Supplementary Table S2. Diagnostic performance of intermediate to high CRB-65 scores for predicting adverse clinical outcomes.

	CRB-65 Cut-off	Sensitivity (95% CI)	Specificity (95% CI)	PPV (95% CI)	NPV (95% CI)
**Inpatient mortality**					
All patients	1	47 (39-55)	76 (72-80)	41 (35-47)	80 (78-83)
	2	10 (6-16)	98 (96-99)	64 (45-80)	76 (75-77)
	3	1 (0-4)	100 (99-100)	50 (6-94)	74 (74-74)
Normal CXR	1	52 (32-71)	72 (64-79)	24 (17-33)	90 (85-93)
	2	7 (1-24)	99 (96-100)	50 (13-87)	86 (85-88)
	3	0 (0-13)	100 (98-100)	-	86 (86-86)
Abnormal CXR	1	46 (37-55)	79 (73-83)	50 (42-57)	76 (73-79)
	2	11 (6-18)	97 (95-99)	67 (45-83)	71 (69-72)
	3	1 (0-4)	100 (98-100)	50 (6-94)	69 (69-69)
**NIV requirement**					
All patients	1	71 (60-81)	30 (26-34)	14 (13-16)	86 (82-90)
	2	95 (88-99)	4 (3-6)	14 (13-15)	84 (65-94)
	3	99 (94-100)	0 (0-1)	14 (14-14)	50 (6-94)
Normal CXR	1	89 (52-100)	33 (26-40)	6 (5-8)	98 (90-100)
	2	100 (66-100)	2 (1-6)	5 (5-5)	-
	3	100 (66-100)	0 (0-2)	5 (5-5)	-
Abnormal CXR	1	69 (57-79)	29 (24-34)	18 (16-20)	80 (74-86)
	2	95 (87-99)	5 (3-8)	18 (17-19)	81 (60-93)
	3	99 (93-100)	0 (0-2)	18 (18-19)	50 (6-94)
**Intubation / ICU admission**					
All patients	1	77 (65-87)	31 (27-35)	12 (11-14)	92 (87-95)
	2	100 (95-100)	5 (3-7)	12 (11-12)	-
	3	-	-	-	-
Normal CXR	1	83 (36-100)	32 (26-40)	4 (3-6)	98 (91-100)
	2	100 (54-100)	2 (1-6)	3 (3-3)	-
	3	100 (54-100)	0 (0-2)	3 (3-3)	-
Abnormal CXR	1	76 (63-86)	30 (25-35)	16 (14-18)	88 (82-92)
	2	100 (94-100)	6 (4-9)	15 (15-16)	-
	3	-	-	-	-

CI: confidence interval; CXR: chest X-ray; ICU: intensive care unit; NIV: non-invasive ventilation; NPV: negative predictive value; PPV: positive predictive value.
